# Serious and Life-Threatening Pregnancy-Related Infections: Opportunities to Reduce the Global Burden

**DOI:** 10.1371/journal.pmed.1001324

**Published:** 2012-10-09

**Authors:** Courtney A. Gravett, Michael G. Gravett, Emily T. Martin, Jeffrey D. Bernson, Sadaf Khan, David S. Boyle, Sophia M. R. Lannon, Janna Patterson, Craig E. Rubens, Matthew S. Steele

**Affiliations:** 1 Global Alliance to Prevent Prematurity and Stillbirth, Seattle Children's Hospital, Seattle, Washington, United States of America; 2 Department of Obstetrics and Gynecology, University of Washington School of Medicine, Seattle, Washington, United States of America; 3 School of Pharmacy, Wayne State University, Detroit, Michigan, United States of America; 4 Program for Appropriate Technology in Health, Seattle, Washington, United States of America; 5 Department of Pediatrics, University of Washington School of Medicine, Seattle, Washington, United States of America

## Abstract

Michael Gravett and colleagues review the burden of pregnancy-related infections, especially in low- and middle-income countries, and offer suggestions for a more effective intervention strategy.

**Summary Points**

- Pregnancy-related infections are one of the leading causes of maternal mortality worldwide, with the burden falling disproportionately on low- and middle-income countries.
- Such infections can be categorized into four different syndromes occurring at distinct times during pregnancy: puerperal sepsis, septic abortion, pyelonephritis/urosepsis, and rapidly progressive soft tissue infections.
- Bundled packages of interventions targeted at these different syndromes should be integrated into antenatal care at two time points: initiation of antenatal care and onset of labor.
- Affordable, point-of-service diagnosis and treatment, coupled with improved descriptive and microbiologic data, are urgently needed to further reduce deaths from pregnancy-related infections.

## Introduction

Infection is an important, potentially preventable, and yet often overlooked cause of maternal mortality and morbidity as well fetal and neonatal well-being. Puerperal sepsis, narrowly defined by the World Health Organization (WHO) as infection of the genital tract occurring any time between the rupture of membranes or labor and the 42nd day postpartum, is the third leading cause of maternal mortality, responsible for 10%–12% of maternal deaths [1]. Deaths due to puerperal sepsis disproportionately occur in low- and middle-income countries (LMICs). The risk of death from puerperal sepsis is 2.7-fold higher in Africa, 1.9-fold higher in Asia, and 2.1-fold higher in Latin America than in developed countries [2].

However, the impact of infection during pregnancy upon maternal, fetal, and neonatal mortality and morbidity is much greater than that attributed to puerperal sepsis alone—the impact includes deaths and disability from other infections such as urinary tract, soft tissue, and abortion-related infections. Septic abortion, for example, is a significant contributor to maternal infectious mortality, responsible for about 50,000 maternal deaths annually [3]. Intrauterine infection is a risk factor for postpartum endometritis (PPE) as well as a significant contributor to preterm birth, which is now recognized as the second biggest cause of newborn deaths [4]. The burden of pregnancy-related infections goes far beyond puerperal sepsis alone. A useful term that incorporates all of these different infections is “life-threatening pregnancy-related infections,” and we use this term in our article.

This Policy Forum article aims to highlight opportunities for screening and appropriate treatment of life-threatening pregnancy-related interventions. Our policy recommendations are based on a review of the published literature on the disease burden associated with life- threatening pregnancy-related infections and the most common causal pathogens in LMICs. In reviewing maternal infections, when they occur in pregnancy, and their associated pathogens, we sought to identify appropriate strategies for detecting infections and providing effective interventions to help reduce the burden of these mostly preventable maternal, fetal, and neonatal deaths and disabilities. We pay particular attention to Africa and Asia, two regions that together account for 80% of global maternal mortality and where puerperal sepsis accounts for 10%–12% of all maternal deaths [5].

**Figure 1:**
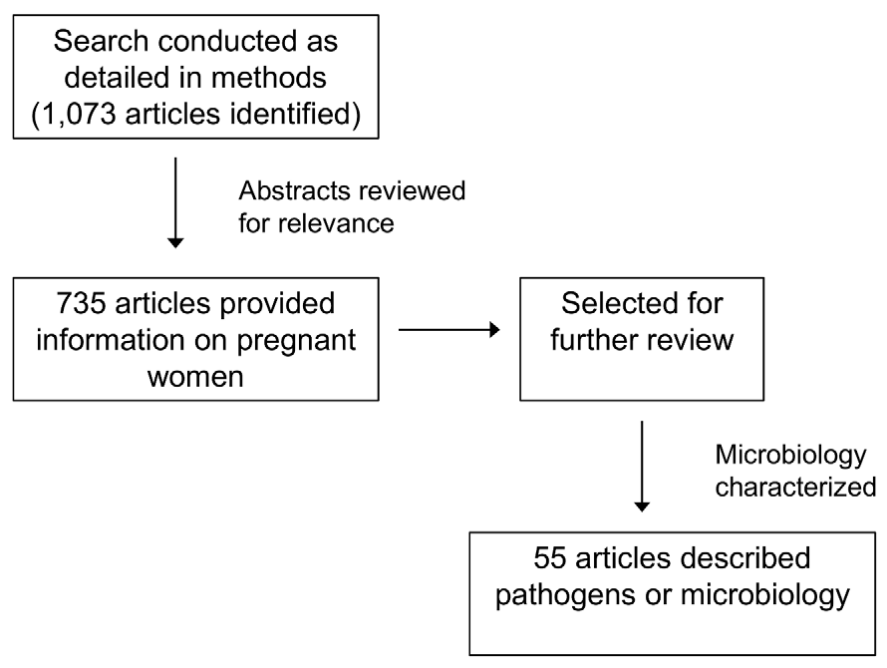
Literature review strategy. Relevant studies from the years 1980 to 2012 were identified by searching PubMed, Medline, Embase, and WHO publications. The search strategy used was “([infectious syndrome]) AND (Africa OR Asia OR India OR Thailand OR Bangladesh)”; the infectious syndromes included in our search were those that cause the most morbidity and mortality: puerperal sepsis (PPE, chorioamnionitis), septic abortion, pyelonephritis or urosepsis, and soft tissue infections (necrotizing fasciitis, group A streptococcal infection, and methicillin-resistant staphylococcal infection). All titles and abstracts were reviewed, and those including etiology, prevalence, or pregnancy were selected for further analysis.

Infections not related specifically to pregnancy (e.g., HIV, curable sexually transmitted infections, tuberculosis, and malaria) are also important contributors to maternal mortality and morbidity. These important non-pregnancy-related infections are beyond the scope of “life-threatening pregnancy-related infections” included in our analysis here, but they indirectly contribute to puerperal sepsis. For example, the risk of puerperal sepsis is increased among women with HIV [6], while chlamydial and gonoccocal infections are important causes of septic abortion [7]. Maternal infections, both pregnancy-related and non-pregnancy-related, remain an important and potentially preventable cause of maternal mortality, especially in LMICs. The review methods are summarized in Figure 1.

## Distinct Maternal Infectious Syndromes

**Figure 2:**
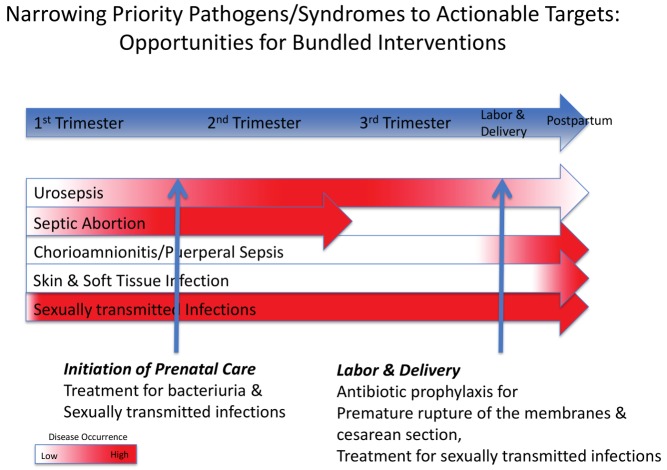
Timing of four clinical syndromes and their pathogens, and potential opportunities for “bundled” diagnostic–therapeutic interventions. See Table 2 for further details of the interventions.

Our review identified four clinical syndromes, and microbes associated with them, that appear to be responsible for most cases of life-threatening pregnancy-related infections in LMICs: (1) puerperal sepsis (chorioamnionitis and PPE), (2) pyelonephritis/urosepsis, (3) septic abortion, and (4) skin and soft tissue infections (SSTIs) (Table 1). Each of these occurs at a distinct time during pregnancy, providing opportunities for screening and prevention, which we discuss further below (Figure 2; Table 2). Although we found only 55 studies that included microbial culture results, many of which had design weaknesses (e.g., inadequate culture techniques, inappropriate culture sites), we identified several commonalities between the four syndromes discussed above that could be exploited to develop an effective intervention strategy. A consistent pattern of genital-urinary pathogens associated with the four syndromes discussed above was identified that was similar to that of pathogens reported in high-income countries (Table 1). Most serious maternal infections were polymicrobial and involved both facultative and anaerobic bacteria indigenous to the lower genital tract or rectum, with the exception of pyelonephritis/urosepsis, which was associated with a single pathogen in most cases, usually *Escherichia coli*. Sexually transmitted pathogens, including *Neisseria gonorrhoeae* and *Chlamydia trachomatis*, were important pathogens in septic abortion.

**Table 1:** Common syndromes associated with life-threatening maternal infections and the pathogens most frequently associated with these.

Clinical Syndrome	Commonly Associated Microorganisms
**Puerperal sepsis (chorioamnionitis/endometritis)**	Mycoplasmataceae
	Enterobacteriaceae
	Group B streptococcus
	*Staphylococcus aureus*
	*Gardnerella vaginalis*
	*N. gonorrhoeae*
	*C. trachomatis*
**Pyelonephritis/urosepsis**	*E. coli*
	Enterobacteriaceae
	*Enterococcus* spp.
	Group B streptococcus
	*S. saprophyticus*
**Septic abortion**	*Bacteroides* spp.
	*Clostridium* spp.
	*C. trachomatis*
	*N. gonorrhoeae*
	Enterobacteriaceae
	*S. aureus*
	Group A streptococcus
	Group B streptococcus
**Soft tissue infection**	Group A streptococcus
	*S. aureus*
	*Bacteroides* spp.
	*Clostridium* spp.
	Enterobacteriaceae

**Table 2:** Interventions for reducing serious morbidities and mortalities from pregnancy-related infections.

Intervention	Timing	Details	Limitations
Treatment of bacteriuria	Antenatal care	Antibiotics with coverage for *E. coli*	In LMICs diagnosis can be challenging in the absence of culture facilities or other testing
Treatment of sexually transmitted infections	Antenatal care	Treatment for gonococcal and chlamydial infection	In LMICs diagnosis can be challenging in the absence of culture facilities or other testing, and women are often asymptomatic
Antibiotic prophylaxis for PROM and pPROM	Labor and delivery	Broad spectrum antibiotics	Effectiveness of treatment is dependent on local patterns of antibiotic resistance
Antibiotic prophylaxis for cesarean section	Labor and delivery, postpartum	Broad spectrum antibiotics	Effectiveness of treatment is dependent on local patterns of antibiotic resistance

pPROM, preterm premature rupture of membranes; PROM, premature rupture of membranes.

Diagnosis and management of these infections in LMICs is based upon recognition of the clinical syndrome because culture is often not available and treatment cannot be delayed while waiting for culture results. This syndromic approach to managing pregnancy-related infections relies on early recognition of clinical signs and symptoms and the ability to aggressively intervene when infection is suspected. The WHO Integrated Management of Pregnancy and Childbirth guidelines [8] (provided in Text S1) outline diagnostic and treatment options relevant to LMICs.

### Puerperal Sepsis

The reported incidence of puerperal sepsis in developing countries ranges from 0.1% to 10%, but the true incidence is difficult to determine [1]. The wide incidence range likely reflects discrepancies in diagnostic criteria, lack of access to health care, and incomplete case ascertainment. Case fatality rates for puerperal sepsis as high as 30% to 50% have been reported in LMICs [9].

Most puerperal sepsis in reviewed reports occurred as two distinct clinical syndromes: (1) intrapartum uterine infection preceding or during labor (clinical chorioamnionitis), and (2) early postpartum infection following birth (PPE), as summarized in Table 3. These infections usually represent ascending infections from the lower genital tract. They share a common microbial etiology, many risk factors, and clinical features (fever and uterine tenderness), and both are associated with neonatal infectious sequelae. Chorioamnionitis is a common complication of pregnancy that may lead to serious adverse maternal, fetal, and neonatal outcomes. Chorioamnionitis is usually the result of ascending polymicrobial infection from the lower genital tract into the chorioamnion. The resulting infection triggers a maternal and fetal inflammatory response and may lead to infection of the fetus, premature rupture of the membranes, preterm labor, and neonatal complications. PPE is an infection of the uterus that occurs in 5% of all vaginal births and 10% of cesarean deliveries in high-income countries [10], but is inconsistently reported in LMICs. PPE is usually caused by ascending infections from bacteria indigenous to the lower genital tract flora, although exogenous, sexually transmitted microorganisms including *N. gonorrhoeae* and *C. trachomatis* may also cause PPE [11]. Cesarean section is an important risk factor for PPE.

**Table 3:** Puerperal sepsis: chorioamnionitis.

Clinical Syndrome	Incidence	Risk Factors	Complications	Etiology	Prevention
Chorioamnionitis	1% to 4% of pregnancies in HICs [20]; no data in LMICs	PROM, preterm PROM, prolonged rupture of membranes, prolonged labor, multiple vaginal examinations, alcohol/tobacco use, nulliparity, vaginal colonization with GBS, bacterial vaginosis, internal fetal monitoring, meconium-stained fluid, epidural anesthesia [21]	Serious maternal fetal and neonatal complications; maternal bacteremia (in 5%–10% of women with IAI); increased risk of dysfunctional labor, postpartum hemorrhage, and postpartum infections; increased risk of neonatal sepsis, pneumonia, respiratory distress, and death [22]; increased risk of long-term neuro-developmental delays and cerebral palsy in HICs [23],[24]	Most often result of ascending infection from lower genital tract; in HICs, over 65% of positive amniotic fluid cultures involve at least two organisms [22]; genital mycoplasmas are most commonly isolated bacteria; other common organisms include gram-negative anaerobes and gram-positive cocci; no data in LMICs	Depends on recognizing risk factors including PROM; maternal prophylactic antibiotic use following preterm PROM is associated with significant reduction (relative risk 0.66, 95% CI 0.46–0.96) in chorioamnionitis [25]; no evidence that vaginal irrigation with chlorhexidine reduces risk of chorioamnionitis, although one meta-analysis suggested small reduction in PPE [26]
PPE	5% of vaginal births and 10% of cesarean deliveries in HICs; inconsistent data in LMICs	Cesarean delivery, prolonged labor with ruptured membranes, chorioamnionitis, multiple vaginal examinations, retained products of conception, unhygienic conditions, vaginal GBS colonization, bacterial vaginosis [27]	Severe cases may lead to serious complications including bacteremia, peritonitis, pelvic thromobolitis, and endotoxic shock [28]	In HICs, Mycoplasmataceae, Enterobacteriaceae, group B *streptococcus*, *S. aureus*, *G. vaginalis*, *N. gonorrhoeae*, *C. trachmatis*, *S. pyogenes*; in LMICs, *S. aureus*, *Klebsiella* spp., *Psuedomonas* spp., *E. coli*, *Proteus* spp., *N. gonorrhoeae*, *S. pneumoniae*, and *Salmonella* spp. have been isolated [29]	Antibiotic prophylaxis for elective and non-elective cesarean section reduces the risk of PPE by up to 77% [30]; vaginal cleansing with chlorhexidine has been proposed, but the data are mixed and there is not sufficient evidence to support vaginal antisepsis [31],[32]

HICs, high-income countries; GBS, group B streptococcus; IAI, intra-amniotic infection; PROM, premature rupture of membranes.

### Pyelonephritis/Urosepsis

Acute pyelonephritis is one of the most common severe complications of pregnancy, occurring in 1%–2% of all pregnancies [12] and may result in serious maternal and fetal morbidity if not diagnosed and treated appropriately. Pregnant women are at increased risk of acute pyelonephritis due to normal anatomic and physiologic changes that occur during pregnancy and make the urinary tract system more vulnerable to infection. Pyelonephritis occurs when bacteria normally present in the vagina and distal urethra ascend the urethra and colonize the upper urologic system [13],[14]. Asymptomatic bacteriuria (ASB) commonly precedes pyelonephritis, and screening and treatment of ASB is a recommended and effective practice to prevent the development of pyelonephritis in pregnancy [15]. Table 4 summarizes the risk factors, complications, and etiology of pyelonephritis.

**Table 4:** Pyelonephritis/urosepsis.

Incidence	Risk Factors	Complications	Etiology	Prevention
In HICs, 1% to 2% of all pregnancies [33]; data in LMICs are incomplete, but suggest a similar incidence, with studies reporting rates from 0.145% to 1.3% [34]–[36]	Pyelonephritis is commonly preceded by ASB: ASB is present in 2.5% to 15% of pregnancies, and advances to pyelonephritis in 20% to 40% of untreated women [37],[38]; other risk factors include nulliparity, sickle cell anemia, and history of pyelonephritis, urinary tract malformations, or renal calculi [13]	Serious maternal and fetal complications including anemia (occurs in 25% of women), bacteremia (occurs in 12% to 20% of women), hypertension, transient renal failure, respiratory insufficiency, sepsis/septic shock, increased risk of preterm labor and preterm birth, low birth weight [13]	Results from ascending infection of the urinary tract system; *E. coli* is responsible for the majority of cases—isolated in 80% to 90% of cases; other organisms isolated from patients include *Klebsiella* spp., *Enterococcus* spp., *P. mirabilis*, and *S. saprophyticus*	Screening and follow-up antibiotic treatment for ASB during pregnancy has been shown to reduce the risk of pyelonephritis by 80% [15]

HICs, high-income countries.

### Septic Abortion

WHO estimates that 21.6 million unsafe abortions take place every year, with 98% of these occurring in developing countries, where abortion is either illegal or accessibility to legal abortions is limited [3]. Unsafe abortion is a major contributor to maternal mortality, accounting for 68,000 maternal deaths worldwide, 5 million hospital admissions, and 1.7 million cases of secondary infertility in developing countries each year [3]. Septic abortion is a common complication of unsafe abortion. Septic abortion has been reported to be responsible for up to 88% of complications from unsafe abortions [16] and is responsible for most deaths due to abortion complications in LMICs [17]. The risk of septic abortion increases as gestation progresses. Many cases go unreported or are diagnosed too late because women are reluctant to seek care following clandestine abortions. In LMICs, sexually transmitted pathogens, especially *N. gonorrhoeae* and *C. trachomatis*, are an important cause of septic abortion (see Table 5 for other risk factors and complications). When women do seek a provider before or after an abortion, providers should take advantage of the opportunity to intercept women for screening and treatment of infections.

**Table 5:** Septic abortion.

Incidence	Risk Factors	Complications	Etiology	Prevention
In HICs, less than 1% of abortions are complicated by infection; in LMICs the incidence of septic abortion is variable, reported at from 4.8% to 22.6%, but true incidence is hard to ascertain due to underreporting	In HICs where abortion is safely and legally performed, septic abortion is extremely rare; in LMICs, septic abortion is much more common due to unsafe, unhygienic procedures that are often illegal and performed by untrained individuals [16]; other risk factors include retained products of conception, preexisting sexually transmitted infection (especially chlamydia or gonorrhea), and insertion of tools, chemicals, or soaps into the uterus [39]	Septic abortion is associated with a high risk of life-threatening complications including death (case fatality rates reported at from 6% to 21% in LMICs) [40]–[42], septic shock, hepato-renal failure, peritonitis, septicemia, anemia, and pelvic inflammatory disease; septic abortion is also a major contributor to secondary infertility and ectopic pregnancy [7]	In HICs and LMICs, most septic abortion cases are polymicrobial, reflecting coliform and vaginal microflora and are anaerobic, including *E. coli*, *Klebsiella* spp., *S. fecalis*, *S. aureus*, *Pseudomonas aeruginosa*, and *P. mirabilis* [43]; the sexually transmitted pathogens *C. trachomatis* and *N. gonorrhoeae* are also implicated [7]; in LMICs, *Clostridium tetani* is frequently reported as a cause of septic abortion due to unhygienic procedures [17]	At the time of the procedure women should receive a risk assessment for sexually transmitted infections, with follow-up screening and treatment for infection [39]; antibiotic prophylaxis (prophylactic doxycycline reduces the risk of postabortal endometritis by 42% [7])

HICs, high-income countries.

### Skin and Soft Tissue Infections

SSTIs are a rare complication of pregnancy, but are associated with high mortality and morbidity. SSTIs are classified as either uncomplicated or complicated. Uncomplicated SSTIs are superficial infections such as simple abscesses, mastitis, and cellulites, and are associated with a low risk of life- or limb-threatening complications. Complicated SSTIs, including streptococcal cellulites, clostridial myonecrosis, and necrotizing fasciitis, are characterized by involvement of deep soft tissue and are associated with a high risk of life- or limb-threatening complications that typically require surgical intervention [18] (Table 6). Many SSTIs originate at infected wound sites following surgical procedures or traumatic vaginal births.

**Table 6:** Complicated SSTIs (necrotizing fasciitis, cellulites, and myositis) during pregnancy.

Incidence	Risk Factors	Complications	Etiology	Prevention
Extremely rare in HICs [44]; no data for LMICs	Obesity, diabetes, trauma to the skin or soft tissue, unhygienic procedures, contamination of wound or surgical site [45]	Complications are severe and include septicemia and tissue necrosis; high mortality rates associated with necrotizing fasciitis (up to 50%) [44]	Most (62% to 75%) infections are polymicrobial and result from introduction of endogenous bacteria, such as *S. aureus*, *Ps. aeruginosa*, or *Streptococcus* spp. [46],[47], into sterile sites; when monomicrobial infection occurs, *S. aureus* and group A streptococcus are most commonly implicated [48]; no data for LMICs	Hygienic practices; patient education regarding risk factors and symptoms of postpartum infections

HICs, high-income countries.

## Discussion

Our landscape review found significant gaps in knowledge about the burden, etiology, and diagnosis of maternal life-threatening infections and about how best to prevent and treat these infections in women in low-resource settings. There are few reliable epidemiologic data from LMICs about the incidence of life-threatening pregnancy-related infections and their microbial etiology. Many births occur at home or in community clinics, which may lead to ascertainment bias that may overestimate maternal infections (e.g., individuals are disproportionately referred to facilities and infections are then reported) or underestimate them (e.g., individuals die at home or infections go unreported). Studies from LMICs usually report only those patients that either deliver in a facility or are referred to a facility because of infection.

There were also significant gaps in the descriptive microbial etiology of pregnancy-related infections in LMICs. Microbial data are lacking, or inadequately assessed, in many studies. Obtaining appropriate samples for bacterial culture is time-consuming, technically challenging, and expensive. The upper genital tract and endometrium are relatively inaccessible; cultures obtained through the cervix, a frequently reported method, are contaminated with indigenous lower genital tract microflora and may not represent upper genital tract pathogens. Anaerobic bacteria and genital mycoplasmas, both important causes of chorioamnionitis and endometritis, have not been consistently sought or identified in most studies from LMICs because of the difficulty in culturing these organisms. Treatment of life-threatening pregnancy-related infections is therefore largely empiric, based upon clinical diagnosis and syndromic management of maternal infections [8].

The lack of reliable population-based incidence estimates and accurate microbiological data hinder development of efficacious treatment and prevention programs that may be readily implemented at the community level (e.g., screening for asymptomatic bacteria). Despite the challenges in detecting and preventing pregnancy-related infections, a few meaningful commonalities and trends point toward a targeted strategy for reducing serious pregnancy-related infections.

Most notably, in our review, we identified a limited number of discrete clinical syndromes that account for most life-threatening pregnancy-related infections in LMICs, each of which tends to cluster at a certain point in gestation. This provides the opportunity for focused programs in screening and intervention across the continuum of care that lend themselves to “bundled” diagnostic–therapeutic interventions, either singly or as combinations of multiple testing and treatment regimens (Figure 2). We have recognized two such points for bundled interventions that target maternal infections and coincide with when a pregnant woman might seek health care: the initiation of prenatal care and the onset of labor. These two time points represent the periods of highest disease occurrence for life-threatening maternal infections, and therefore the times when screening, diagnosis, and treatment are most critical.

Women should be screened for ASB and curable sexually transmitted infections at the time of their first antenatal visit to prevent pyelonephritis and reduce septic abortion. Screening and treatment of ASB by urinalysis or culture at the first prenatal contact substantially reduces the risk of subsequent pyelonephritis and is recommended for all pregnant women. Screening for *N. gonorrhoeae* and *C. trachomatis* at the first prenatal visit or prior to a scheduled abortion has been shown to reduce post-abortion infections in developed countries [11]. Simple bundled interventions during labor and delivery also afford the opportunity to reduce maternal infections. A single dose of prophylactic antibiotics given at the time of cesarean delivery reduces the risk of PPE. Similarly, prophylactic antibiotics given following preterm premature rupture of the membranes reduces maternal and neonatal infectious morbidity in developed countries and should also be efficacious in LMICs. Finally, although rapidly progressive soft tissue infections occur sporadically, and do not lend themselves to a systematic preventive strategy, education of providers and patients as part of a bundled package at delivery may lead to earlier recognition and prompt treatment, with improved survival.

To reduce the burden of life-threatening pregnancy-related infections, significant limitations in our knowledge and delivery of care need to be directly addressed. There is a great need for comprehensive studies in LMICs exploring the epidemiology, risk factors, and microbiology of life-threatening maternal infections. These studies should include infections occurring at the level of the community and household, where most births occur, as well as facility-based programs. The lack of readily available microbiologic data points to the need for rapid, reliable point-of-care adjunctive diagnostic tests. The attributes of an ideal diagnostic test have been outlined by WHO as an acronym, ASSURED, standing for affordable, sensitive, specific, user-friendly, robust and rapid, equipment-free, and deliverable to those in need [19]. Except for detection of bacteriuria and detection of certain sexually transmitted infections, tests meeting these criteria do not currently exist for life-threatening maternal infections.

Regional differences in pathogens and antimicrobial susceptibility and resistance patterns should be specifically addressed in high-burden LMICs. Ongoing microbiologic surveillance surveys are also necessary to identify shifts in microbial flora or emergence of microbial resistance. Without such data, women will continue to be treated inappropriately and experience potentially preventable mortality and morbidity.

## Supporting Information

Text S1 **Diagnostic and treatment guidelines for serious maternal infections in LMICs.**
(PDF)

## Abbreviations

ASB: asymptomatic bacteriuria
LMICs: low- and middle-income countries
PPE: postpartum endometritis
SSTIs: skin and soft tissue infections
WHO: World Health Organization
