# Supplementary material for: Serious and Life-Threatening Pregnancy-Related Infections: Opportunities to Reduce the Global Burden
Source: PLoS Med. 2012 Oct 9;9(10):e1001324. doi: 10.1371/journal.pmed.1001324 (PMC3467240; doi:10.1371/journal.pmed.1001324)
Supplement: Text S1 — Diagnostic and treatment guidelines for serious maternal infections in LMICs. (PDF) [file pmed.1001324.s001.pdf]

Supplemental Appendix. Diagnostic and Treatment Guideline for Serious Maternal Infections in LMIC*

Syndrome	Signs & Symptoms Typically Present	Signs & Symptoms Sometimes Present	Diagnosis- Gold Standard	Diagnosis- Other	Treatment	Follow-up
**Acute Pyelonephritis**	• Dysuria<br>• Spiking fever/chills<br>• Increases frequency and urgency of urination<br>• Abdominal pain<br>• Costovertebral anle pain	• Retropublic/suprapubic pain<br>• Loin/pain tenderness<br>• Tenderness in ribcage<br>• Anorexia<br>• Nausea/vomiting	• Mid-stream clean-catch urine culture<br>• Antibiotic sensitivity testing if possible	• Urine dipstick (leukocyte esterase or nitrate reductase)<br>• Urine Gram stain	• Initiate treatment for shock, if present<br>• Start IV infusion, infuse IV fluids at 150 mL/hour<br>• Treat with anitbiotic appropriate for organism<br>• If urine culture is unavailable treat with ampicillin* 2 g IV every 6 hours *plus* gentamicin 5 mg/kg body weight IV every 24 hours	• Re-evaluate antibiotic regime if no improvement after 72 hours<br>• Continue treatment until woman is free of fever for 48 hours, then give amoxicillin 1 g by mouth 3 times/day to complete 14 days of treatment**
**Septic Abortion**	• Foul-smelling vaginal discharge in first 22 weeks<br>• Fever<br>• Uterine tenderness<br>• Cervical motion tenderness	• Lower abdominal pain<br>• Rebound tenderness<br>• Prolonged bleeding<br>• Purulent cervical discharge	• Relies on clinical signs and symptoms and should be considered in any woman of reproductive age presenting with vaginal bleeding, abdominal pain and fever.	• Pelvic exam to visualize vaginal or cervical laceration and visible retained products of conception<br>• Positive pregnancy test indicates a current or recent pregnancy	• Immediately initiate antibiotic therapy: Ampicillin 2 g IV every 6 hours *plus* gentamicin 5 mg/kg body weight IV every 24 hours *plus* metronidazole 500 mg IV every 8 hours<br>• Removal of retained products of conception<br>• Fluid resuscitation and support therapy as needed to manage septic shock	• Continue antibiotic therapy until woman is free of fever for 48 hours
**Chorioamnionitis (Intra-amniotic infection)**	• Fever/chills<br>• Foul-smelling watery discharge after 22 weeks<br>• Abdominal pain	• History of amniotic fluid loss<br>• Tender uterus<br>• Rapid fetal heart rate<br>• Light vaginal bleeding	• Amniocentesis followed by culture of the amniotic fluid	• Based on clinical signs of inflammation including the presence of maternal fever (>38C) with the presence of 2 other signs including maternal leukocytosis (>15,000 cells/mm3), maternal tachycardia (>100bpm), fetal tachycardia (>160bpm), uterine tenderness and foul smelling/purulent amniotic fluid	• Give antibiotics until delivery: Ampicillin 2 g IV every 6 hours *plus* gentamicin 5 mg/kg body weight every 24 hours<br>• Deliver baby as soon as possible:<br>• If cervix is favourable, induce labor with oxytocin<br>• If cervix is unfavorable, ripen cervix with prostaglandins and infuse oxytocin or perfrm caesarean section	• If vaginal delivery occurs discontinue antibiotics, but if c-scetion occurs contibue antibiotics and give metronidazole 500 mg IV every 8 hours unitl fever-free for 48 hours<br>• Monitor for development of puerperal sepsis, and if suspected give antibiotics<br>• If neonatal sepsis is supected, perform blood culture and give antibiotics

Syndrome	Signs & Symptoms Typically Present	Signs & Symptoms Sometimes Present	Diagnosis- Gold Standard	Diagnosis- Other	Treatment	Follow-up
**Puerperal Sepsis/Endometritus**	• Fever/chills<br>• Lower abdominal pain<br>• Purulent, foul smelling lochia<br>• Tender uterus	• Light vaginal bleeding<br>• Shock	• Relies on recognition of clinical signs and symptoms including maternal fever (>38C), pain, uterine tenderness, cervical motion tenderness, tachycardia, leukocytosis and unusual or foul smelling discharge	• Culture of endometrial cavity may confirm diagnosis of sepsis, but is problematic because cultures obtained transvaginally or transcervically may be contaminated	• Immediately initiate antibiotic therapy:<br>• Ampicillin 2 g IV every 6 hours *plus* gentamicin 5 mg/kg body weight IV every 24 hours *plus* metronidazole 500 mg IV every 8 hours<br>• Provide support therapy as needed<br>• If present, remove retained placental fragments<br>• If uterus is necrotic and septic perform hysterectomy	• Continue antibiotic therapy until woman is free of fever for 48 hours If no improvement, and signs of general peritonitis are present, perfom laparotomy to drain pus
**Cellulitis & Necrotizing Fasciitis**	• Painful and tender wound<br>• Erythema and oedema beyond edge of incision<br>• Intense pain<br>• Unexplained fever	• Hardened wound<br>• Purulent discharge<br>• Reddened area around wound	• Relies on recognition of clinical signs and symptoms, especially pain, redness and other signs of inflammation<br>• Direct observation of wound site to look for redness, heat or tenderness as well as tissue separation<br>• Wound site culture	• Imaging studies	• *If infection does not involve deep tissue:* Ampicillin 500 mg by mouth 4 times/day for 5 days *plus* metronidazole 400 mg by mouth 3 times/day for 5 days; if fluid or pus is present, open and drain would; remove infected skin or subcutaneous sutures and decride wound<br>• *If infection involves deep tissues:* Penicillin G 2 million units IV every six hours *plus* gentamicin 5 mg/kg body weight IV every 24 hours *plus* metronidazole 500 mg IV every eight hours; wide surgical debridement is mandatory#	• For necrotizing fasciitis, once woman is fever free for 48 hours, give ampicillin 500 mg IV 4 times/day for 5 days *plus* metronidazole 400 mg by mouth 3 times/day for 5 days

*All guidelines taken from IMPAC puiblication: Managing Complications in Pregnancy and Childbirth: A guide for midwives and doctors

** Treatment with penicillins not recommended in high income countries with high prevalence of E. coli resistance. Use 2nd or 3rd genreation cephalosporin

Appropriate surgical debridement is mandatory (average patient requires 3 debridements). Survival with antibiotic therapy alone is rare.
